# Integrated human carotid plaque transcriptomics and macrophage perturbation link *BCAP31* to endoplasmic reticulum stress, inflammation, and apoptosis

**DOI:** 10.3389/fcell.2026.1970662

**Published:** 2026-10-23

**Authors:** Yunlong Wang, Bin Lin, Shanjiang Chen, Jianjian Yang, Anwu Huang

**Affiliations:** Department of Cardiology, Wenzhou Central Hospital, Wenzhou, China

**Keywords:** apoptosis, atherosclerosis, *BCAP31*, carotid plaque, endoplasmic reticulum stress, inflammation, macrophage

## Abstract

**Background:**

Atherosclerotic plaque progression couples chronic inflammation to dysregulated cell survival, but the cellular context and regulatory role of endoplasmic reticulum (ER)-associated death responses in human carotid disease remain incompletely resolved.

**Methods:**

We integrated paired bulk transcriptomes from 32 carotid plaque and macroscopically intact tissue pairs, laser-captured macrophages from five ruptured and six stable plaques, whole-plaque transcriptomes from 27 intraplaque-hemorrhage and 16 non-hemorrhage lesions, and single-cell RNA sequencing from three patients with matched atherosclerotic core and proximal adjacent tissue. BCAP31 was evaluated by siRNA-mediated depletion in oxidized low-density lipoprotein (oxLDL)-exposed THP-1-derived macrophages, with PCR, immunofluorescence, ELISA, apoptosis assays, and 4-phenylbutyrate treatment.

**Results:**

Paired bulk analysis showed coordinated increases in apoptosis, ferroptosis, pyroptosis, necroptosis, inflammasome, inflammatory-response, and TNF/NF-κB programs. Ruptured plaque macrophages showed increases in ferroptosis, pyroptosis, and necroptosis scores, whereas intraplaque-hemorrhage plaques showed broader activation across all prespecified programs. Patient-level single-cell pseudobulk analysis localized a directionally consistent increase in BCAP31 to macrophages, although the estimate did not remain significant after transcriptome-wide correction in the three-patient dataset. Cross-sectional state ordering placed higher BCAP31 expression toward plaque-enriched HLA-C1QC, APOC1-SPP1-remodeling, and SPP1-lipid-loaded macrophage states, while predicted macrophage-to-vascular-wall SPP1 signaling increased in all three patient pairs. BCAP31 was also increased in paired plaque, rupture-macrophage, and intraplaque-hemorrhage cohorts and belonged to a plaque-associated coexpression module. In macrophages derived from THP-1 cells and treated with oxLDL, BCAP31 knockdown increased ER-stress transcripts, nuclear DDIT3, inflammatory cytokine expression and release, and Annexin V-positive apoptosis. The chemical chaperone 4-phenylbutyrate attenuated these effects.

**Conclusion:**

Human plaque transcriptomics and macrophage perturbation support a stress-adaptive role for BCAP31 in ER-stress-associated inflammation and apoptosis. Increased tissue expression and the adverse effects of depletion indicate that plaque-associated upregulation may reflect a compensatory response.

## 1 Introduction

Advanced atherosclerotic plaques are not simply lipid deposits; they are chronically inflamed tissues in which macrophage accumulation, extracellular-matrix remodeling, cell loss, and defective clearance determine whether a lesion remains stable or develops a necrotic, rupture-prone phenotype (Bentzon et al., 2014; Moore and Tabas, 2011). Macrophage death is context dependent. In early lesions, apoptosis followed by efficient efferocytosis may constrain cellularity, whereas in advanced plaques inefficient clearance converts apoptotic-cell accumulation into secondary necrosis and expansion of the necrotic core (Linton et al., 2016). Human lesions associated with acute coronary syndromes show prominent ER stress, and experimental deletion of the ER-stress effector CHOP/DDIT3 reduces macrophage apoptosis and plaque necrosis in advanced murine atherosclerosis (Myoishi et al., 2007; Thorp et al., 2009). These observations place cell death and inflammatory amplification within the same disease process rather than in parallel, unrelated pathways.

The unfolded protein response initially restores proteostasis by reducing protein synthesis, increasing chaperone capacity, and promoting disposal of misfolded proteins. When stress is sustained or cannot be resolved, the same network can engage inflammatory signaling and apoptosis (Hetz, 2012; Ron and Walter, 2007). This transition is particularly relevant to plaque macrophages, which encounter oxidized lipids, hypoxia, cholesterol loading, cytokines, and defective efferocytosis. Although apoptosis, necroptosis, pyroptosis, and ferroptosis are mechanistically distinct forms of regulated cell death (Galluzzi et al., 2018), transcriptomic signatures in diseased tissue often reflect overlapping stress and inflammatory programs. Work on tumor immunity further illustrates how the mode of cell death shapes immune responses through danger-signal release (Lu et al., 2024). Patient-matched tissue contrasts and patient-level single-cell aggregation can help distinguish lesion-associated expression from changes in cell composition, while independent instability cohorts establish the phenotype in which a signal occurs.

BCAP31 encodes B-cell receptor-associated protein 31 (BAP31), an abundant ER membrane protein involved in protein trafficking, ER quality control, and communication between the ER and mitochondria (Quistgaard, 2021). BAP31 biology is unusually dependent on molecular state. Uncleaved full-length BAP31 can restrain Fas-associated mitochondrial apoptosis, whereas caspase cleavage generates p20BAP31, which promotes ER calcium release, mitochondrial fission, and cytochrome-c mobilization (Nguyen et al., 2000; Breckenridge et al., 2003; Iwasawa et al., 2011). BAP31 also participates in transmission of ER-stress death signals through the CDIP1-BAP31 complex (Namba et al., 2013). Thus, increased BCAP31 transcript abundance could reflect an adaptive attempt to preserve ER function, an expanded stressed-cell compartment, or a context in which pro-death cleavage becomes more likely. Transcript abundance alone cannot resolve these possibilities.

Previous integration of bulk and single-cell atherosclerosis datasets has identified immune-associated candidates including CD36, S100A10, and CSNK1A1 (Chen et al., 2024). To examine how lesion-associated expression relates to cellular stress responses, we combined four independently phenotyped human carotid plaque datasets with a patient-paired analytical framework. The study first mapped death-associated programs in paired plaque and adjacent tissue, tested their relation to rupture and intraplaque hemorrhage, localized candidate expression by single-cell pseudobulk and macrophage-state analysis, and evaluated network context and predicted intercellular communication. BCAP31 emerged from this cross-layer screen and was subsequently tested in oxidized low-density lipoprotein (oxLDL)-exposed THP-1-derived macrophages using siRNA, quantitative PCR, immunofluorescence, ELISA, and Annexin V/propidium iodide flow cytometry. The central question was not whether BCAP31 was merely elevated in plaque, but whether its perturbation shifted the ER-stress-associated inflammation-apoptosis axis in a direction consistent with a pathogenic or compensatory role.

## 2 Materials and methods

### 2.1 Study design and public transcriptomic datasets

Publicly available expression data were obtained from the NCBI Gene Expression Omnibus (GEO) (Edgar et al., 2002). GSE43292 was used for discovery and contained 32 patient-matched carotid plaques and macroscopically intact tissue samples (Ayari and Bricca, 2013). GSE41571 provided laser-microdissected plaque macrophages from 5 patients with ruptured plaques and 6 with stable plaques (Lee et al., 2013). GSE159677 contained single-cell RNA-sequencing profiles from atherosclerotic core (AC) and proximal adjacent (PA) tissue from three matched patients (Alsaigh et al., 2022). GSE163154 included whole-plaque samples from 27 patients with intraplaque hemorrhage (IPH) and 16 without IPH (Jin H. et al., 2021). Accession metadata, tissue labels, pairing variables, and clinical endpoints were audited before analysis. The independent unit was the patient or matched tissue pair; cells, clusters, communication edges, and ordering bins were treated as lower-level observations.

### 2.2 Bulk-transcriptome preprocessing and differential expression

Processed expression matrices and platform annotations were retrieved from GEO. For GSE41571, the positive GCRMA-processed signal-intensity columns labeled “raw” in the deposited matrix_NORMALIZED_RAW_ABSCALL file were log2-transformed before probe selection and analysis; these are processed intensities, not CEL-file measurements. Supplementary columns were matched to GEO samples by comparison with their normalized-ratio profiles. The ratio-scale series matrix was not used as log2 expression. The deposited log-scale expression matrices were used for GSE43292 and GSE163154. Probes were mapped to gene symbols with platform-specific annotation resources, and when multiple probes mapped to the same gene the probe with the greatest across-sample variance was retained. GSE43292 was analyzed with a paired design containing tissue and patient terms. GSE41571 was modeled with rupture status, scaled age, and sex, and GSE163154 was modeled with IPH status. Gene-level and module-level contrasts were fitted with limma using empirical-Bayes moderation with robust and trend options (Ritchie et al., 2015). Effect estimates are reported as plaque minus intact tissue, ruptured minus stable macrophages, or IPH minus non-IPH plaque. Benjamini–Hochberg false-discovery rates (FDRs) were calculated within each prespecified testing family.

### 2.3 Cell-death and inflammatory-program scoring

The seven gene sets were HALLMARK_APOPTOSIS, GOBP_FERROPTOSIS, REACTOME_PYROPTOSIS, GOBP_NECROPTOTIC_PROCESS, REACTOME_INFLAMMASOMES, HALLMARK_INFLAMMATORY_RESPONSE, and HALLMARK_TNFA_SIGNALING_VIA_NFKB. Exact gene membership is recorded in cell_death_gene_set_ledger.csv in the accompanying analysis source files. Sample-level scores were computed by single-sample gene-set enrichment using GSVA with the ssGSEA method, minimum and maximum set sizes of 3 and 500, alpha of 0.25, and score normalization enabled (Hänzelmann et al., 2013). The inflammatory-response and TNF/NF-κB sets were analyzed beside death programs to determine whether apparent death-associated signals were separable from broad inflammatory activation. Contrasts between these scores are module-score differences, not log2 fold changes. Gene-level effect estimates retain the log2 fold-change scale.

### 2.4 Single-cell quality control, integration, and cell-type annotation

The GSE159677 count matrix was separated into six patient-condition samples using the verified barcode suffix map. Genes detected in fewer than 3 cells and cells with fewer than 200 detected genes were removed. Cells were further required to contain 200–4,000 detected genes and less than 10% mitochondrial reads. Doublets were identified independently in each sample with scDblFinder and excluded (Germain et al., 2021). Singlets were log-normalized in Seurat; 3,000 variable genes were selected, data were scaled, and 40 principal components were computed (Stuart et al., 2019). Harmony integration was performed using sample identity, followed by neighbor construction with the first 30 components, clustering at resolution 0.55, and UMAP visualization with seed 20,260,811 (Korsunsky et al., 2019). Major cell classes were assigned by concordance across canonical markers and cluster-level marker programs, then manually audited. Automatic labels were not accepted when marker support was mixed.

### 2.5 Patient-level pseudobulk and macrophage-state analyses

For each major cell class, raw counts were aggregated by patient, condition, and cell type. A comparison was analyzed only when all six patient-condition pseudobulks were available and each contributing pseudobulk contained at least 50 cells. Genes were filtered with edgeR filterByExpr using minimum count 10 and minimum total count 30, normalized by the trimmed mean of M values, and tested in a paired quasi-likelihood model with robust dispersion estimation (McCarthy et al., 2012). Module scores were the mean gene-wise standardized log-counts per million (prior count 1) across the six pseudobulks, requiring at least three mapped genes per set. For each cell-type-program combination, the three within-patient AC-minus-PA differences were tested against zero using a two-sided one-sample t-test (2 degrees of freedom), equivalent to a paired t-test. Global FDR correction covered all 49 combinations of seven retained cell classes and seven programs; within-class FDRs were also retained in source files. Macrophages were reclustered after normalization, selection of 2,500 variable genes, Harmony integration, and clustering at resolution 0.45 using 25 dimensions. States were named from marker programs as inflammatory monocyte, HLA-C1QC, APOC1-SPP1 remodeling, SPP1 lipid-loaded, and low-RNA mixed myeloid. The low-RNA mixed state was retained for descriptive composition plots but excluded from primary state-level inference. State tests required all six pseudobulks and at least 20 cells per patient-condition-state unit.

### 2.6 Cross-sectional macrophage-state ordering

Slingshot was used to place marker-audited macrophage states along cross-sectional expression manifolds (Street et al., 2018). To limit sample-size imbalance, a maximum of 500 cells per patient-condition-state stratum was included. The inflammatory-monocyte state was specified as the starting state; APOC1-SPP1 remodeling and SPP1 lipid-loaded states were specified endpoints of the fitted transcriptional ordering. Two algorithmic branches were retained: inflammatory monocyte to HLA-C1QC to APOC1-SPP1 remodeling, and inflammatory monocyte to SPP1 lipid-loaded. Feature trends were summarized across ordering bins together with patient and condition coverage. For macrophage-state ordering, per-cell program scores were mean log-normalized expression over the mapped genes, rather than ssGSEA scores. The custom ER-stress-associated apoptosis set comprised BCAP31, HSPA5, DDIT3, ATF4, XBP1, ERN1, EIF2AK3, ATF6, CASP3, CASP8, BAX, and BCL2. Because all samples were cross-sectional, the resulting coordinate was interpreted only as transcriptional ordering and not as observed time, lineage tracing, or evidence that one state converts into another.

### 2.7 Predicted cell-cell communication

CellChat analyses were conducted separately for each of the six GSE159677 samples using CellChatDB.human (Jin S. et al., 2021). Eligible major cell classes contained at least 100 cells in every sample; each patient-condition-cell-class stratum was downsampled to no more than 150 cells. Overexpressed genes and interactions were identified with thresholds of 0.1 for the expression proportion and fold change and 0.05 for nominal P values. Communication probabilities were computed with the truncated-mean method (trim 0.1), without population-size weighting, and interactions involving fewer than 50 cells were removed. Pathway and ligand-receptor outputs were summarized as paired AC-minus-PA differences across the three patients. These outputs were treated as computational predictions rather than measurements of ligand binding or pathway activity.

### 2.8 Coexpression network and functional enrichment

Weighted gene coexpression network analysis was performed in GSE43292 using the 5,000 most variable genes together with BCAP31 (Langfelder and Horvath, 2008). Soft-threshold diagnostics used signed bicorrelation across powers 1–10 and 12–20; no tested power reached a scale-free fit of 0.80, and power 20, with the highest fit, was selected. Module construction used a signed Pearson-correlation network, signed topological overlap, minimum module size 30, and merging height 0.25. Network construction used all 64 tissue profiles; module association was tested separately using limma models with patient and tissue terms, followed by empirical-Bayes moderation and Benjamini–Hochberg correction across the nine eigengenes, including the grey unassigned set. Module effects are reported as plaque-minus-intact eigengene differences. BCAP31 module membership and descriptive module-status bicorrelations were retained. Gene Ontology biological-process enrichment for the BCAP31-containing module and for genes whose paired plaque-minus-intact changes correlated with BCAP31 changes was performed with clusterProfiler using Benjamini–Hochberg correction (Wu et al., 2021). Paired-change correlations were Spearman coefficients across 32 patients. The four strongest positive correlates were additionally examined using patient-level scatterplots and leave-one-patient-out correlations.

### 2.9 Candidate prioritization

Candidate screening began with the union of apoptosis, ferroptosis, pyroptosis, and necroptosis genes. The bulk screen required paired-plaque FDR <0.05 and recorded independent rupture and IPH support when nominal P < 0.05 and the effect direction agreed with the paired-plaque result. Candidates supported by all three bulk cohorts entered the original single-cell localization screen. Cellular support required median AC detection of at least 10%, at least 100 A C UMIs, positive AC-minus-PA expression in all three patients, nominal pseudobulk P < 0.05, and positive effects in all three bulk cohorts. Transcriptome-wide pseudobulk FDR was reported separately from this localization criterion. BCAP31 was selected for perturbation after this screen and a qualitative PubMed literature assessment recorded on 11 August 2026. Searches combined gene names or aliases with atherosclerosis and, where relevant, macrophage or cell-death terms. HMOX1, GPX4, and NINJ1 were not pursued because of existing mechanistic literature; SPP1 was retained as cellular context, CASP1 had a different cellular localization, and TSPO lacked a focused death-related rationale. This was a research-priority decision, not a numerical novelty score or proof of absence of prior work. The candidate-level statistics, cellular criteria, search strings, and decisions are retained in the accompanying analysis source files.

### 2.10 Cell culture and oxLDL treatment

THP-1 cells (ATCC, TIB-202) were maintained in RPMI-1640 medium (Gibco, 11,875,093) supplemented with 10% fetal bovine serum (Gibco, 10,099,141) and 1% penicillin-streptomycin (Gibco, 15,140,122) at 37 °C with 5% CO2. Macrophage differentiation was induced with 100 nM phorbol 12-myristate 13-acetate (PMA; Sigma-Aldrich, P8139) for 24 h, followed by 24 h in PMA-free complete medium, a differentiation strategy consistent with established THP-1 macrophage models (Daigneault et al., 2010). Differentiated cells received the indicated oxLDL concentrations for the concentration-response experiment or 50 μg/mL oxLDL for 24 h in subsequent experiments. Cultures were monitored for *mycoplasma*, and only negative cultures were used.

### 2.11 Quantitative real-time PCR

Total RNA was isolated with TRIzol Reagent (Invitrogen, 15,596,026) and reverse-transcribed with PrimeScript RT Master Mix (Takara, RR036 A). Quantitative PCR used TB Green Premix Ex Taq II (Takara, RR820 A), with 95 °C for 30 s, 40 cycles of 95 °C for 5 s and 60 °C for 30 s, and melting-curve analysis. Transcripts measured were BCAP31, HSPA5, ATF4, DDIT3, spliced XBP1 (XBP1s), TNF, IL1B, and IL6, with GAPDH as reference. Relative expression was calculated by the 2^−ΔΔCT^ method (Livak and Schmittgen, 2001). Gene-specific primers were synthesized by Sangon Biotech. Primer sequences are provided in Supplementary Table S1.

### 2.12 Cell-viability assay

Cell viability was measured with Cell Counting Kit-8 (Dojindo, CK04). Differentiated THP-1 cells were exposed to 0, 25, 50, or 100 μg/mL oxLDL for 24 h, incubated with CCK-8 reagent for 2 h, and read at 450 nm. Viability was expressed relative to the untreated control.

### 2.13 siRNA transfection and 4-PBA treatment

Three siRNAs targeting BCAP31 (siBCAP31-1, siBCAP31-2, and siBCAP31-3) and a non-targeting control (siNC) were synthesized by GenePharma (custom siRNA, A01004). Differentiated cells were transfected with 50 nM siRNA using Lipofectamine RNAiMAX (Invitrogen, 13,778,075), and knockdown was assessed by qPCR at 48 h SiBCAP31-2 produced the strongest transcript reduction and was used as siBCAP31 thereafter. Formal experiments comprised siNC + vehicle, siBCAP31 + vehicle, siNC + oxLDL, and siBCAP31 + oxLDL. The 4-PBA experiments comprised siNC + vehicle, siNC + oxLDL, siBCAP31 + oxLDL, and siBCAP31 + oxLDL + 4-phenylbutyrate (4-PBA). 4-PBA (Sigma-Aldrich, SML0309; 5 mM) was added 1 h before oxLDL and maintained during the 24-h exposure.

### 2.14 DDIT3 immunofluorescence

Cells on coverslips were fixed in 4% paraformaldehyde for 15 min, permeabilized with 0.1% Triton X-100 for 10 min, and blocked with 5% bovine serum albumin for 1 h. Cells were incubated overnight at 4 °C with mouse monoclonal anti-CHOP/DDIT3 antibody (Cell Signaling Technology, 2,895; 1:500), followed by Alexa Fluor 488-conjugated goat anti-mouse IgG (Invitrogen, A-11001; 1:2000) for 1 h at room temperature. Nuclei were counterstained with DAPI. Images were acquired using identical settings across groups, and nuclear DDIT3 intensity was quantified in ImageJ using DAPI-defined regions.

### 2.15 Enzyme-linked immunosorbent assay

Culture supernatants were collected after treatment and clarified by centrifugation. TNF-alpha, IL-1beta, and IL-6 were quantified with human Quantikine kits (R&D Systems: DTA00D, DLB50, and D6050B, respectively) according to the manufacturer’s protocols. Absorbance was measured at 450 nm, concentrations were calculated from standard curves, and values were normalized to the corresponding cell number and expressed as pg per 10^6^ cells.

### 2.16 Annexin V/propidium iodide apoptosis assay

Apoptosis was measured with an APC Annexin V/propidium iodide kit (Invitrogen, 88–8,007-74). Adherent and detached cells were collected, washed with cold PBS, resuspended in 1x binding buffer, and stained for 15 min at room temperature in the dark. Early apoptotic cells were Annexin V-positive/PI-negative and late apoptotic cells were Annexin V-positive/PI-positive; total apoptosis was the sum of both populations.

### 2.17 Statistical analysis

The original transcriptomic analyses were performed in R 4.5.1 using limma 3.64.3, GSVA 2.2.1, Seurat 5.4.0, Harmony 2.0.2, scDblFinder 1.22.0, edgeR 4.6.3, Slingshot 2.16.0, CellChat 1.6.1, WGCNA 1.74, and clusterProfiler 4.16.0. The corrected GSE41571 and paired module-association models were fitted with R 4.5.3 and limma 3.62.2; full session information accompanies the code. Bulk samples and patient-level pseudobulks, not individual cells, were the independent units. Limma empirical-Bayes models were used for bulk gene and module tests, edgeR quasi-likelihood models for pseudobulk gene tests, and paired t tests for single-cell module-score differences. Two-sided P values were used, and Benjamini–Hochberg FDRs were calculated across genes within each bulk contrast and each single-cell class, across the seven bulk programs within each cohort, across all 49 major-cell-class-program combinations, across the nine network eigengenes, or across terms within each enrichment analysis. Effect estimates, direction across patient pairs, and adjusted P values were interpreted together; nominal P values were identified explicitly when FDR exceeded 0.05. CellChat and cross-sectional ordering outputs were descriptive and were not assigned causal P values. Experimental analyses were performed in GraphPad Prism 10 and are presented as mean ± SD from three independent experiments. Multiple groups were compared by ordinary one-way ANOVA with Šídák adjustment over three selected comparisons per endpoint. The comparison groups and adjusted P values are specified in the corresponding experimental figure legends. Variance homogeneity was examined using the Brown–Forsythe test. Normality tests were not used to establish distributional assumptions with only three observations per group. P < 0.05 was considered significant.

## 3 Results

### 3.1 Paired carotid plaques show coordinated death-associated and inflammatory remodeling

The 32 matched plaque-intact pairs preserved substantial patient-level structure while separating by tissue state in multidimensional scaling (Figures 1A,B). Paired gene-level modeling identified widespread plaque-associated expression changes (Figures 1C,D). All seven prespecified modules increased in plaque: apoptosis (module-score difference 0.0475, FDR 7.42 × 10^−6^), ferroptosis (0.0471, FDR 2.78 × 10^−5^), pyroptosis (0.0965, FDR 7.42 × 10^−6^), necroptosis (0.0503, FDR 2.79 × 10^−7^), inflammasome (0.1388, FDR 1.40 × 10^−7^), inflammatory response (0.1450, FDR 2.47 × 10^−6^), and TNF/NF-κB signaling (0.1057, FDR 3.64 × 10^−5^) (Figures 1E–J). The overlaps among death-program gene sets were limited relative to their distinct membership, indicating that the coordinated tissue-level increase was not produced by one duplicated gene list (Figure 1K). A cross-program screen then identified genes with reproducible paired effects and support beyond the discovery cohort, including BCAP31 (Figure 1L).

**FIGURE 1:**
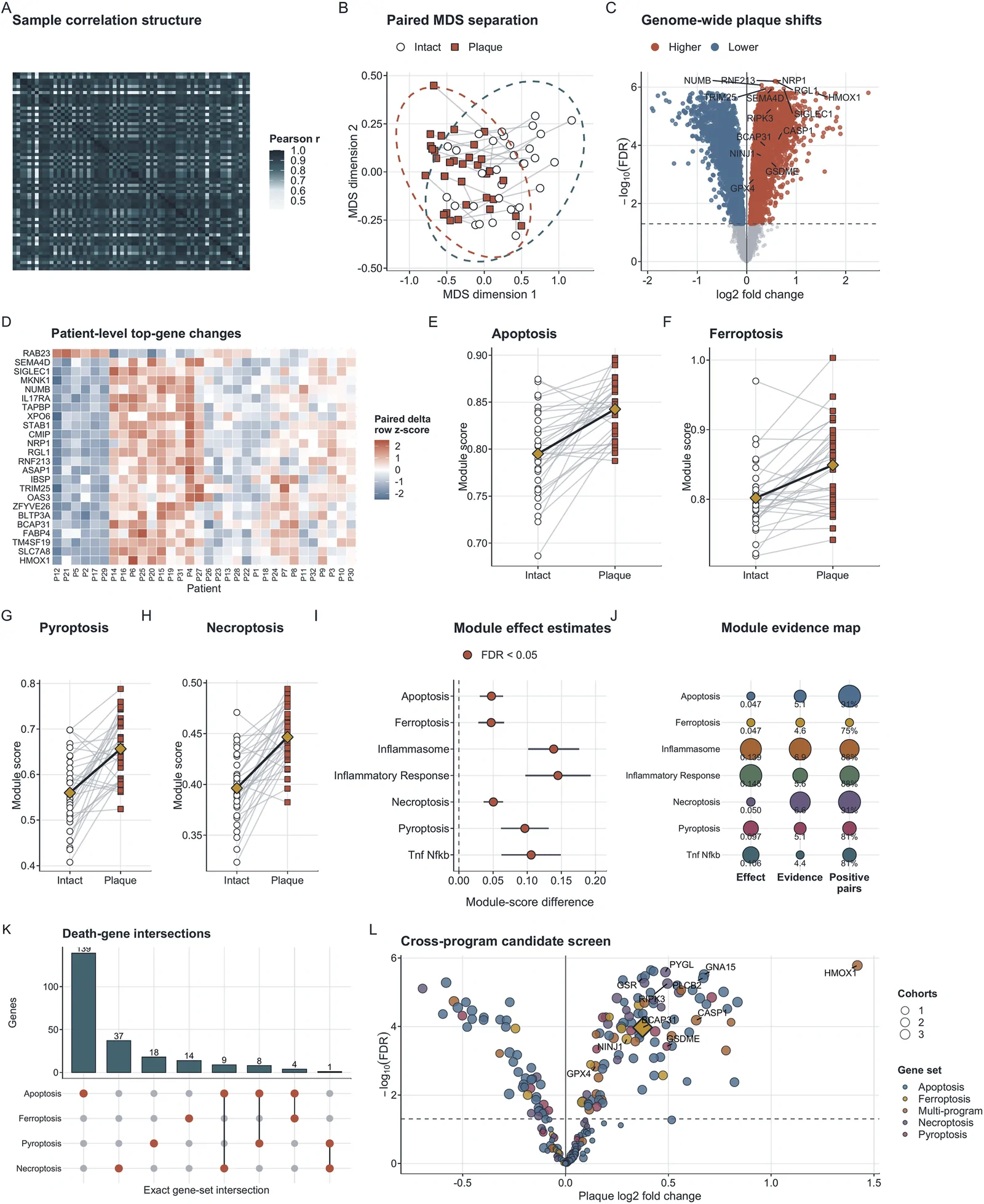
Paired plaque transcriptomic landscape and cell-death program screening. **(A)** Pearson correlation matrix calculated from the 3,000 most variable genes across 32 patient-matched plaque and macroscopically intact carotid tissue pairs. **(B)** Multidimensional scaling of matched samples, with lines connecting tissues from the same patient. **(C)** Genome-wide paired differential-expression results; the horizontal line denotes FDR = 0.05. **(D)** Patient-level standardized expression changes for top plaque-associated genes, calculated as plaque minus matched intact tissue. **(E–H)** Paired patient-level changes in apoptosis, ferroptosis, pyroptosis, and necroptosis module scores, respectively; gold diamonds denote group means. **(I)** Module-score effect estimates and 95% confidence intervals for death-associated and inflammatory programs. **(J)** Integrated module evidence map summarizing effect size, FDR evidence, and the proportion of patient pairs with positive changes. **(K)** Exact intersections among apoptosis, ferroptosis, pyroptosis, and necroptosis gene sets. **(L)** Cross-program candidate screen relating paired plaque effect size to FDR evidence; point size denotes the number of supportive bulk cohorts and *BCAP31* is marked by a gold diamond.

### 3.2 Plaque rupture and intraplaque hemorrhage show different death-program profiles

The rupture cohort comprised five ruptured and six stable plaques, with age and sex distributions shown in Figure 2A. The log2-transformed macrophage transcriptomes showed partial group separation (Figures 2B,C). Ferroptosis, pyroptosis, and necroptosis scores increased in ruptured macrophages, with module-score differences of 0.243 (FDR = 0.00580), 0.189 (FDR = 0.00580), and 0.116 (FDR = 0.0116), respectively. Apoptosis and the remaining inflammatory programs did not reach FDR <0.05 (Figures 2D,E). In contrast, IPH plaques showed broad transcriptomic separation and significant increases in all seven programs, with FDRs from 3.03 × 10^−5^ for apoptosis to 2.64 × 10^−7^ for inflammasome and inflammatory response (Figures 2F–I). Cross-cohort effects therefore identified overlapping but non-identical death-associated profiles in ruptured macrophages and whole-plaque IPH (Figures 2J,K). BCAP31 increased in paired plaque (log2 fold change 0.375, FDR = 1.07 × 10^−4^), ruptured macrophages (0.972, P = 0.00659, FDR = 0.136), and IPH plaque (0.901, FDR = 5.18 × 10^−9^); rupture evidence was nominally significant but did not survive transcriptome-wide correction (Figure 2L).

**FIGURE 2:**
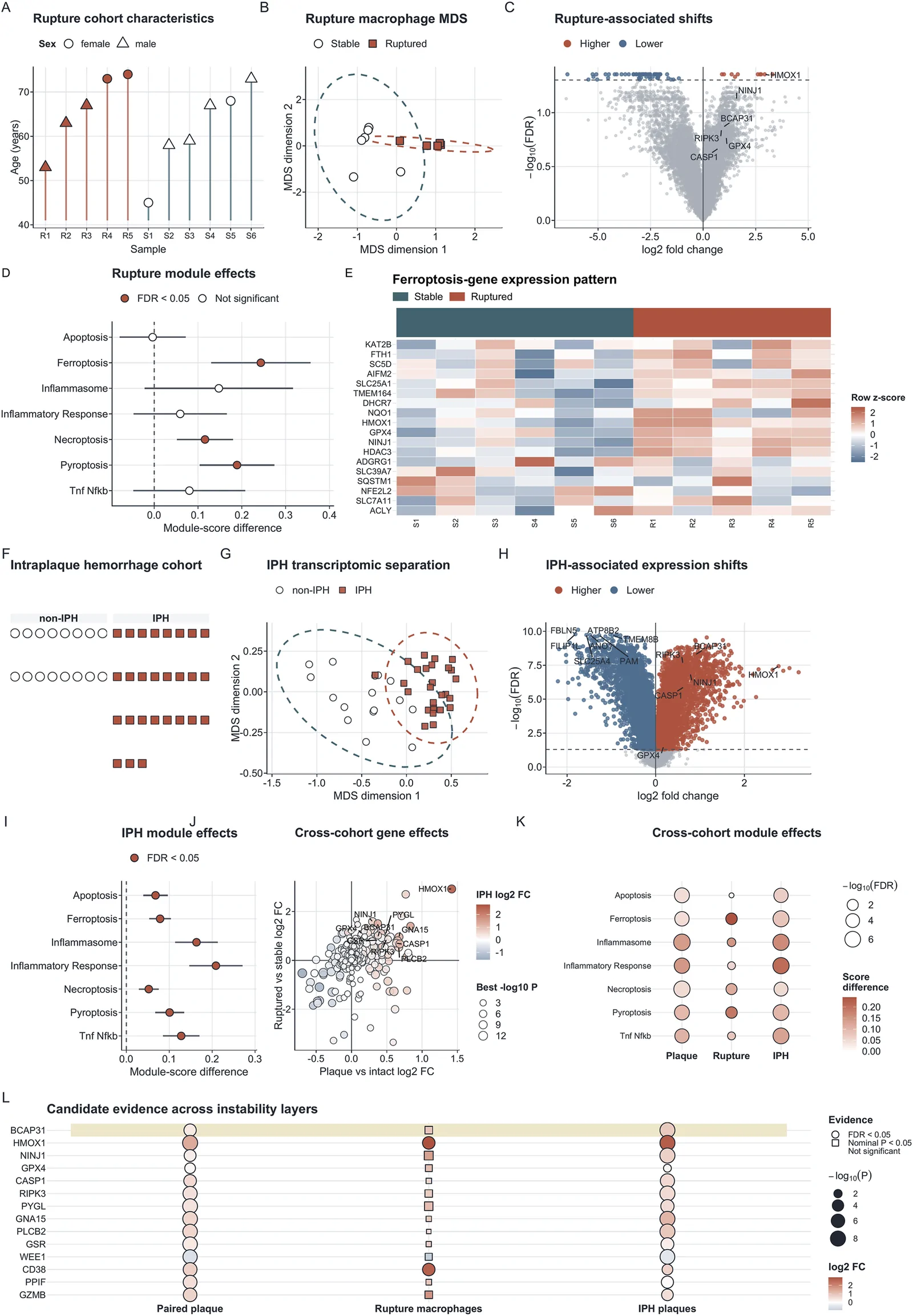
External validation across plaque rupture and intraplaque hemorrhage. **(A)** Age and sex distribution of laser-captured macrophage samples from stable and ruptured carotid plaques in GSE41571. **(B)** Multidimensional scaling of stable and ruptured plaque macrophage transcriptomes. **(C)** Rupture-associated differential-expression results from the corrected log2-intensity analysis, shown without fold-change clipping. **(D)** Effect estimates and 95% confidence intervals for death-associated and inflammatory modules in ruptured versus stable plaque macrophages. **(E)** Row-standardized expression of ferroptosis genes across stable and ruptured plaque macrophage samples. **(F)** Patient-level composition of the GSE163154 intraplaque hemorrhage cohort. **(G)** Multidimensional scaling of intraplaque hemorrhage and non-hemorrhage plaque transcriptomes. **(H)** Genome-wide differential-expression results for intraplaque hemorrhage versus non-hemorrhage plaques. **(I)** Module-score effect estimates and 95% confidence intervals for intraplaque hemorrhage. **(J)** Concordance of gene-level effects across paired plaque, rupture-macrophage, and intraplaque-hemorrhage analyses. **(K)** Cross-cohort matrix of module-score differences and FDR evidence; effects are not log2 fold changes. **(L)** Candidate evidence matrix across the three bulk analyses; symbol shape separates FDR-supported, nominal, and non-significant evidence, and BCAP31 is highlighted.

### 3.3 A patient-audited single-cell atlas resolves plaque cellular composition

Quality control reduced 51,275 input cells to 41,528 retained singlets across the six matched samples, with 2,726–12,343 cells retained per sample (Figures 3A–C). Marker-audited clustering resolved 12 classes: endothelial cells, fibroblasts, macrophages, natural killer cells, pericytes, T cells, vascular smooth muscle cells (VSMCs), B cells, cycling lymphocytes, mast cells, plasma cells, and plasmacytoid dendritic cells (Figures 3D–H). Seven classes met the six-sample and minimum-cell-count criteria for paired pseudobulk testing: endothelial cells, fibroblasts, macrophages, natural killer cells, pericytes, T cells, and VSMCs. Cell counts differed substantially between AC and PA samples, making raw cell-level tests unsuitable for patient inference (Figure 3I). Patient-paired composition analysis showed reproducible but lineage-specific shifts rather than a uniform immune expansion (Figures 3J,K), and the final labels were revised where automatic annotation conflicted with canonical marker support (Figure 3L).

**FIGURE 3:**
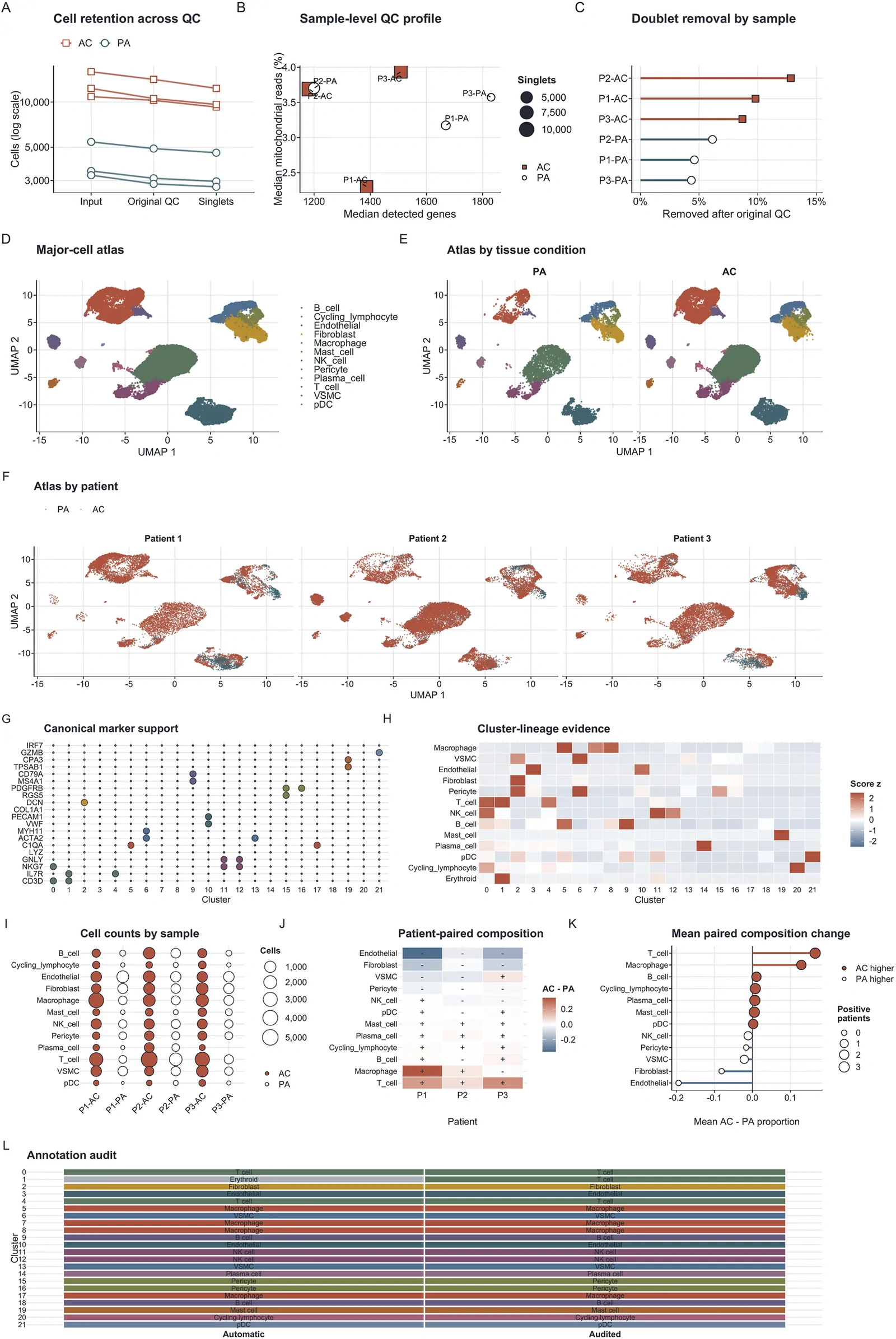
Patient-audited single-cell atlas of carotid plaque tissue. **(A)** Cell retention across input, original quality control, and singlet selection. **(B)** Sample-level detected-gene and mitochondrial-read profiles, with point size indicating retained singlets. **(C)** Doublet-removal fraction by sample. **(D)** UMAP of the full atlas containing 12 annotated cell classes. **(E)** UMAP stratified by paired tissue condition. **(F)** UMAP stratified by patient. **(G)** Canonical marker support across clusters. **(H)** Cluster-lineage marker-score matrix. **(I)** Cell counts by sample and cell class. **(J)** Patient-paired changes in cell-class proportions. **(K)** Mean paired composition changes, with point size indicating the number of patients with AC greater than PA. **(L)** Comparison of automatic and audited cluster annotations. AC, atherosclerotic core; PA, proximal adjacent tissue. Cells are descriptive lower-level observations; patient pairs are the independent units for paired comparisons.

### 3.4 Cell-type-dependent death programs and directional macrophage BCAP31 expression

Patient-level pseudobulk profiles meeting the testing criteria were available for 7 cell classes (Figure 4A). The number of differentially expressed genes varied markedly by lineage (Figure 4B), and the relationship between death-associated and inflammatory changes varied by cell type (Figure 4C), as did individual apoptosis, ferroptosis, pyroptosis, and necroptosis scores (Figures 4D–G). Notably, the macrophage apoptosis score showed no paired increase (mean difference −0.0097, P = 0.967), and no macrophage death module reached FDR <0.05. Fibroblast apoptosis decreased in all three patient pairs and remained significant after global correction (mean difference −0.110, global FDR = 0.0287), illustrating that the coordinated bulk-plaque signal could not be assigned wholesale to every lineage. BCAP31 was broadly detectable but showed its largest patient-level pseudobulk increase in macrophages (log2 fold change 0.756, nominal P = 0.01296, FDR = 0.229); all three patient pairs were positive (Figures 4H–J). Detection and UMI-support audits indicated that this direction was not driven by a single sparse sample (Figure 4K). The integrated gate therefore retained macrophage BCAP31 as a localization signal rather than as a transcriptome-wide significant single-cell association (Figure 4L).

**FIGURE 4:**
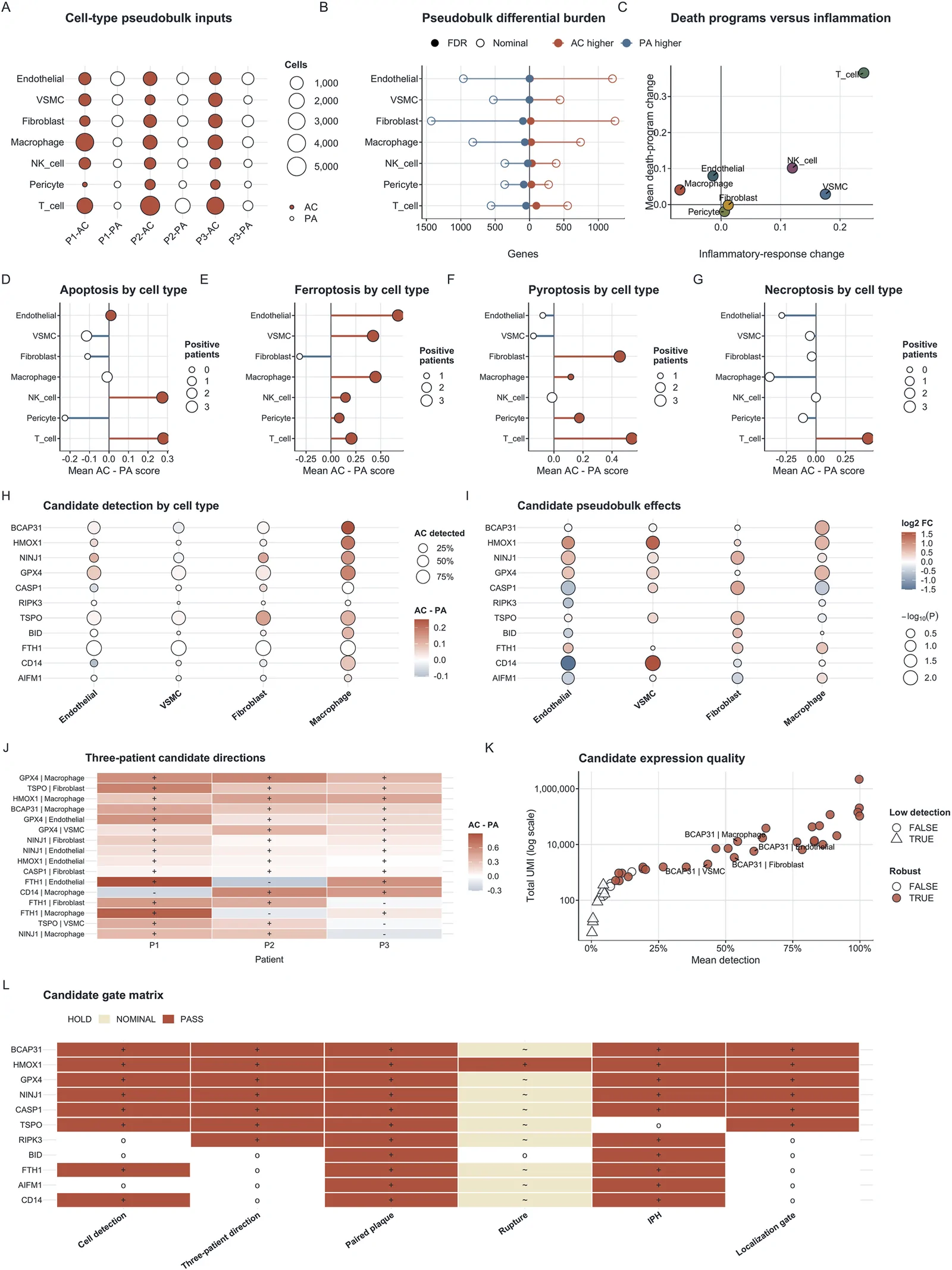
Cell-type localization of death-associated programs and candidate genes. **(A)** Cell counts used to construct patient-level pseudobulk profiles. **(B)** Numbers of nominal and FDR-supported differentially expressed genes by cell class. **(C)** Relationship between mean death-program and inflammatory-response changes. **(D–G)** Patient-paired changes in apoptosis, ferroptosis, pyroptosis, and necroptosis scores, respectively. **(H)** Candidate detection frequencies across major cell classes. **(I)** Candidate pseudobulk effects; point size represents nominal evidence and fill represents log2 fold change. **(J)** Patient-specific candidate-expression directions for prioritized gene-cell-class combinations. **(K)** Detection frequency and total UMI support for candidate-expression quality. **(L)** Evidence gate integrating cellular detection, three-patient direction, paired plaque, rupture, intraplaque hemorrhage, and localization evidence. **(A–G)** concern the 7 cell classes meeting pseudobulk inclusion criteria. Module FDR correction spans all 49 cell-type-program combinations. **(L)** PASS denotes the stated criterion, not necessarily transcriptome-wide significance; NOMINAL denotes P < 0.05 without FDR <0.05.

### 3.5 Plaque macrophages shift toward SPP1-rich remodeling states

Reclustering resolved five macrophage states with distinct marker programs and patient representation (Figures 5A–G). Compared with PA tissue, AC tissue showed mean increases in APOC1-SPP1-remodeling (+0.0740 of macrophages) and SPP1-lipid-loaded states (+0.1722), each positive in all three patient pairs, alongside a decrease in inflammatory monocytes (−0.1713, negative in all three pairs) (Figures 5H–J). BCAP31 was detected across states, with AC-associated increases most evident in HLA-C1QC and inflammatory-monocyte compartments; expression was also high in plaque-enriched SPP1 states, although the PA reference contained very few SPP1-state cells (Figures 5K,L). These expression patterns place BCAP31 within several macrophage remodeling states.

**FIGURE 5:**
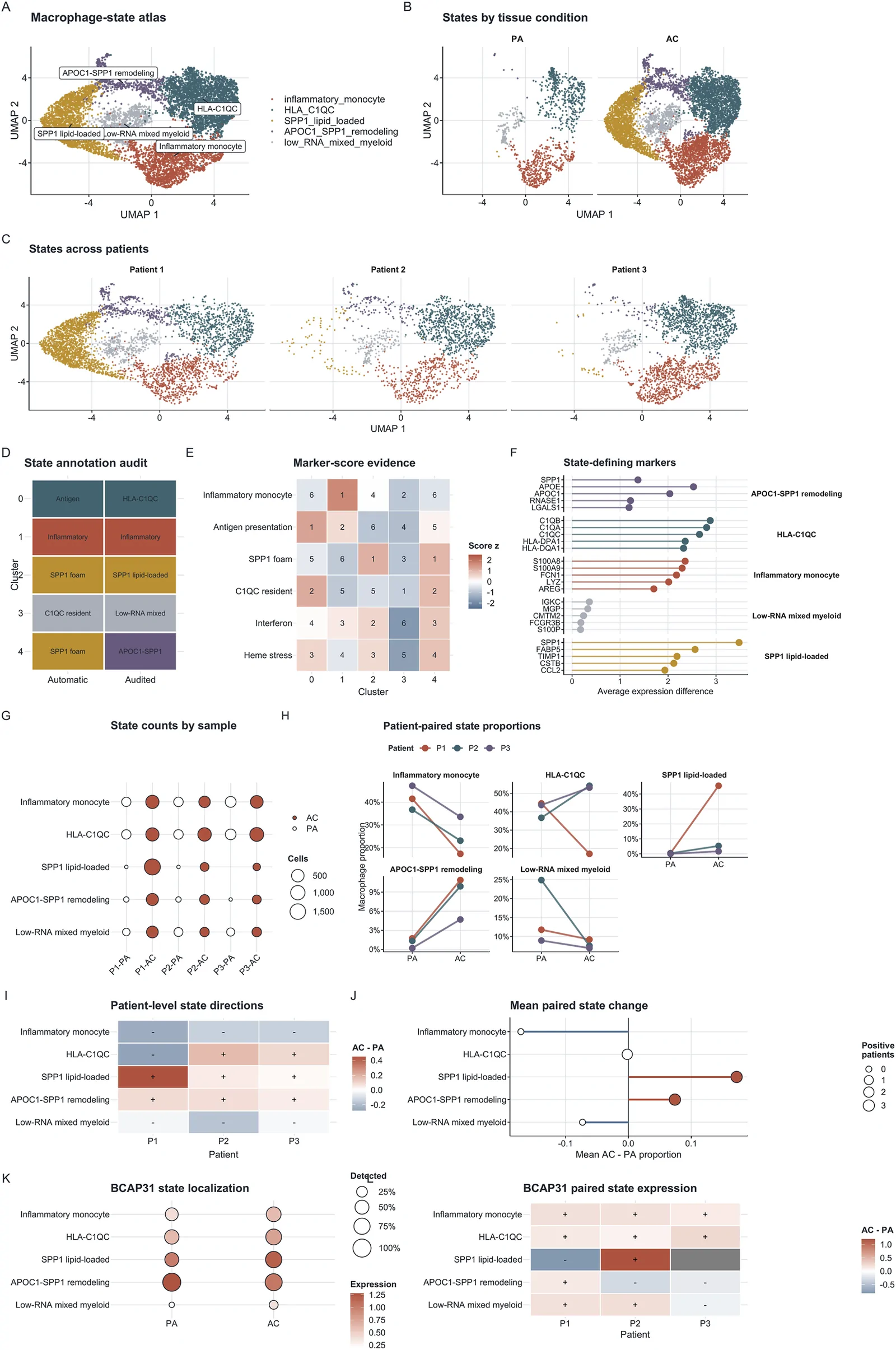
Paired macrophage-state remodeling and *BCAP31* localization. **(A)** UMAP of macrophage states. **(B)** State distribution by tissue condition. **(C)** State distribution across patients. **(D)** Comparison of automatic and marker-audited state labels. **(E)** Marker-program scores across macrophage clusters; numbers indicate program rank within each cluster. **(F)** Top state-defining markers. **(G)** State counts by sample. **(H)** Patient-paired state proportions. **(I)** Patient-specific state-composition directions. **(J)** Mean paired state-composition changes. **(K)** *BCAP31* detection and expression across macrophage states and conditions. **(L)** Patient-paired *BCAP31* expression changes by state. The low-RNA mixed-myeloid state is retained descriptively and excluded from primary state-level inference.

### 3.6 Cross-sectional ordering places *BCAP31* toward plaque-enriched macrophage endpoints

Marker-audited macrophages supported two algorithmically fitted transcriptional branches from inflammatory monocytes: one passing through HLA-C1QC to APOC1-SPP1 remodeling and one with the SPP1-lipid-loaded state as its specified endpoint (Figures 6A–D). AC cells became increasingly dominant toward terminal bins, with both conditions and all three patients represented in earlier portions but reduced sample coverage at some terminal bins (Figures 6E–H). BCAP31 expression rose along both branches, accompanying changes in ER-stress-associated apoptosis and lipid-remodeling features (Figures 6I–L). Because the analysis was cross-sectional and terminal coverage was uneven, these trends support transcriptional placement, not temporal progression or conversion of inflammatory monocytes into SPP1 macrophages.

**FIGURE 6:**
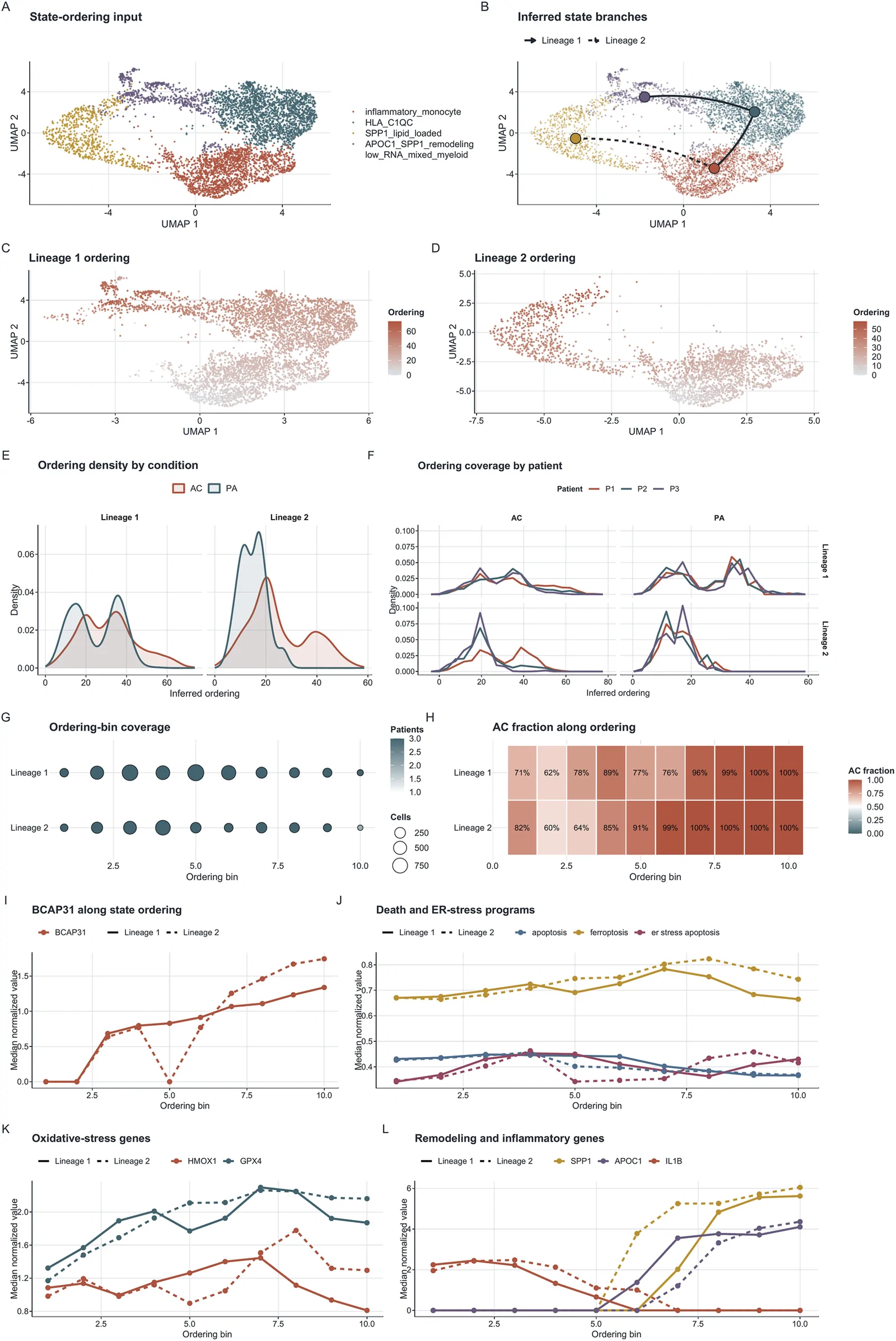
Cross-sectional macrophage-state ordering and death-program context. **(A)** Macrophage-state input used for ordering. **(B)** Algorithmically inferred transcriptional branches connecting inflammatory monocytes with HLA-C1QC and APOC1-SPP1 remodeling states or with the SPP1 lipid-loaded state. **(C,D)** Lineage-specific inferred ordering on the macrophage UMAP. **(E)** Ordering-density distributions by tissue condition. **(F)** Patient coverage across conditions and branches. **(G)** Cell and patient coverage across ordering bins. **(H)** AC fraction across ordering bins. **(I)** BCAP31 expression along both branches. **(J)** Apoptosis, ferroptosis, and ER-stress-associated apoptosis scores along ordering, calculated as mean log-normalized expression of mapped genes. **(K)** HMOX1 and GPX4 expression along ordering. **(L)** SPP1, APOC1, and IL1B expression along ordering. This cross-sectional ordering is exploratory and does not represent observed time or establish lineage causality.

### 3.7 Predicted macrophage-to-wall communication increases in plaque core

Balanced per-sample CellChat analyses showed condition-dependent changes in the number and summed probability of predicted interactions (Figures 7A–C). Several pathways changed consistently within patient pairs, with macrophage outgoing communication showing prominent remodeling (Figures 7D–F). Predicted SPP1 signaling from macrophages to endothelial cells, fibroblasts, and VSMCs increased in all three patients; mean paired probability differences were 0.0447, 0.1204, and 0.0851, respectively (Figures 7G,H). MIF, FN1, and COLLAGEN axes showed additional sender-receiver changes that varied by cell class (Figures 7I–K). Replication plots separated directions supported in all three pairs from sparse or patient-specific predictions (Figure 7L). These predictions identify SPP1-rich macrophage interactions for subsequent experimental investigation.

**FIGURE 7:**
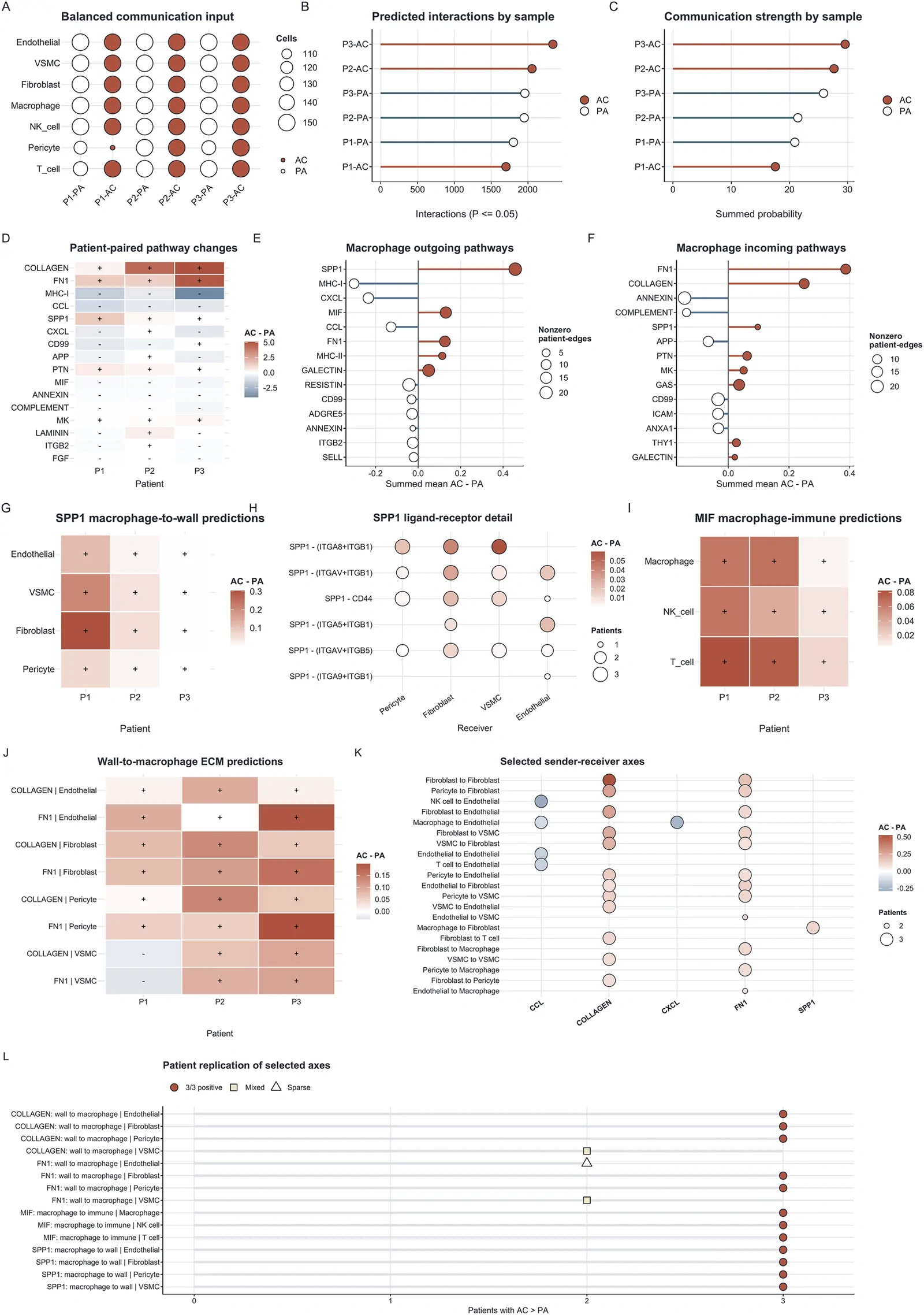
Patient-paired predicted intercellular communication in carotid plaque tissue. **(A)** Balanced cell-class input for CellChat analysis. **(B)** Number of predicted interactions by sample. **(C)** Summed communication probability by sample. **(D)** Patient-paired pathway changes. **(E,F)** Macrophage outgoing and incoming pathway changes. **(G)** Predicted SPP1 signaling from macrophages to vascular-wall cell classes. **(H)** SPP1 ligand-receptor detail. **(I)** Predicted MIF signaling from macrophages to immune receivers. **(J)** Predicted FN1 and COLLAGEN signaling from wall cell classes to macrophages. **(K)** Selected sender-receiver pathway axes. **(L)** Patient replication of selected pathway directions. CellChat outputs are computational predictions and do not establish physical signaling or causality.

### 3.8 Cross-cohort and network integration prioritize *BCAP31* as an adaptive-stress candidate

Cross-cohort effects and patient-level cellular criteria supported BCAP31 prioritization (Figures 8A–D). Its bulk effects were positive in all three cohorts, while the rupture and macrophage pseudobulk findings were nominally significant but not significant after FDR correction. In GSE43292, BCAP31 belonged to the 1,999-gene turquoise module, whose eigengene increased in plaque in the patient-adjusted model (paired difference 0.148, FDR = 1.58 × 10^−7^); BCAP31 module membership was 0.817 (Figures 8E–G). The module was enriched for immune and cytokine-related processes, whereas genes whose paired changes correlated with BCAP31 highlighted lysosomal organization and intracellular-pH context (Figures 8H–J). The four strongest positive correlates were TALDO1, GSDME, CELSR1, and SLC3A2 (Spearman rho = 0.914–0.927); their leave-one-patient-out coefficients remained between 0.905 and 0.944. Cellular and literature-informed selection criteria are distinguished from statistical significance in the integrated evidence matrix (Figures 8K,L).

**FIGURE 8:**
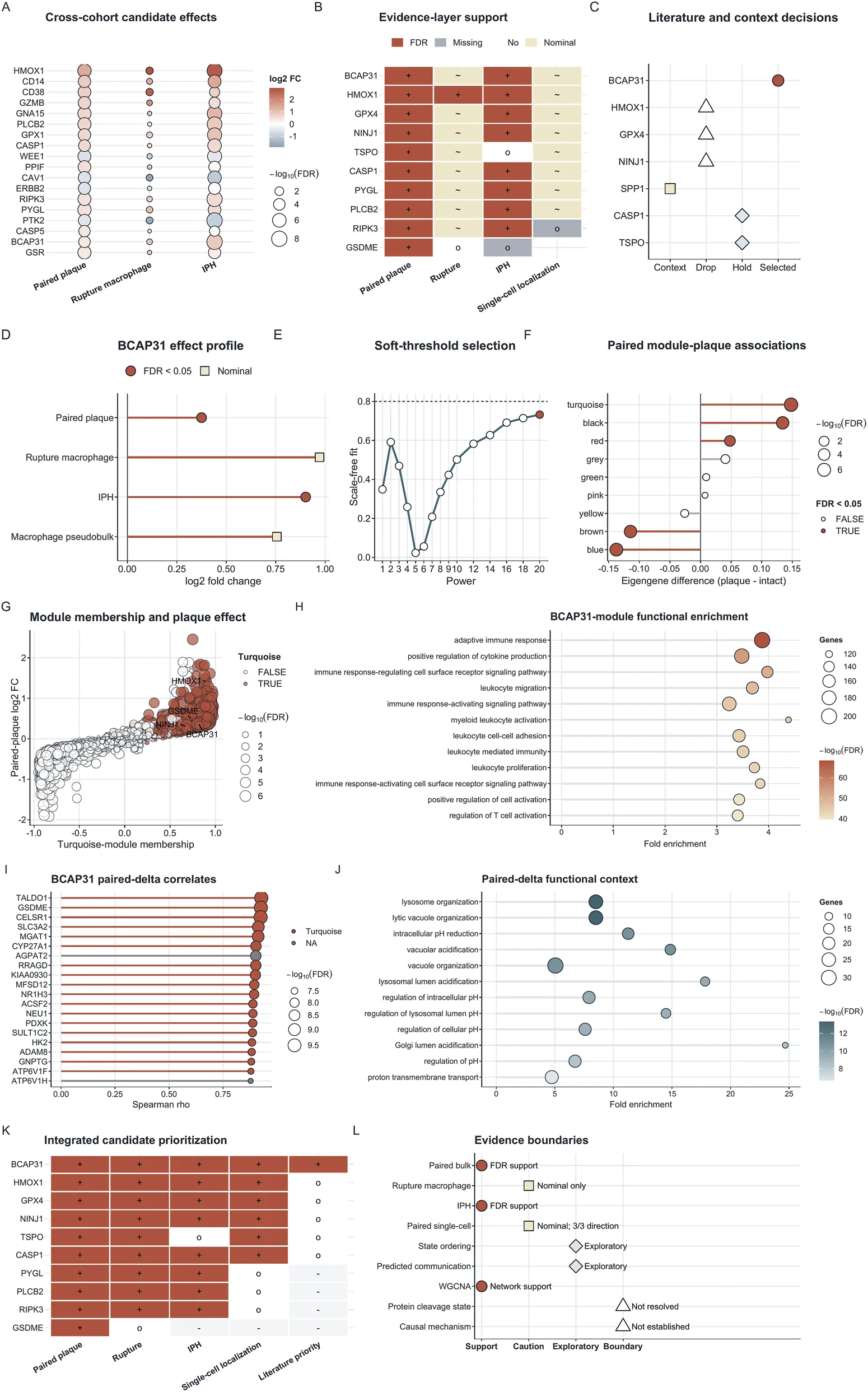
Integrated evidence and prioritization of BCAP31. **(A)** Cross-cohort candidate effect estimates. **(B)** Evidence-layer matrix distinguishing FDR-supported, nominal, non-significant, and unavailable results; single-cell status uses the selected cell-class pseudobulk P value and FDR, not the localization gate. **(C)** Qualitative literature and cellular-context decisions. **(D)** BCAP31 effects across bulk and macrophage pseudobulk analyses. **(E)** Soft-threshold diagnostics; the dashed line denotes the target scale-free fit of 0.80, which was not reached. **(F)** Plaque-minus-intact module eigengene differences from patient-adjusted models, with FDR correction across nine eigengenes. **(G)** Turquoise-module membership versus paired-plaque gene expression effect. **(H)** Functional enrichment of the BCAP31-containing module. **(I)** Genes whose within-patient expression changes correlate with BCAP31 changes across 32 pairs. **(J)** Functional enrichment of the correlated genes. **(K)** Criterion-level candidate matrix; literature priority is a qualitative selection decision, not a statistical or numerical novelty score. A dash denotes a criterion without a recorded assessment. **(L)** Evidence-status summary. BCAP31 rupture and macrophage pseudobulk associations are nominally significant but not significant after transcriptome-wide FDR correction.

### 3.9 *BCAP31* depletion amplifies oxLDL-induced ER stress in THP-1-derived macrophages

OxLDL produced a concentration-dependent loss of viability and induction of *HSPA5* and *DDIT3*. At 50 μg/mL, viability was reduced while both ER-stress transcripts were strongly increased; this concentration was retained for the perturbation experiments (Figures 9A–C). All three siRNAs reduced *BCAP31* mRNA, and siBCAP31-2 produced the strongest knockdown (Figure 9D). In the four-group experiment, oxLDL increased *HSPA5*, *ATF4*, *DDIT3*, and *XBP1s* relative to vehicle controls, and *BCAP31* depletion further increased each transcript under oxLDL challenge (Figures 9E–H). Nuclear DDIT3 immunofluorescence showed the same ordering: signal increased with oxLDL and was highest after combined *BCAP31* depletion and oxLDL exposure (Figures 9I,J). *BCAP31* depletion also increased selected ER-stress readouts under vehicle conditions, consistent with a basal role in ER homeostasis rather than an effect restricted to oxLDL.

**FIGURE 9:**
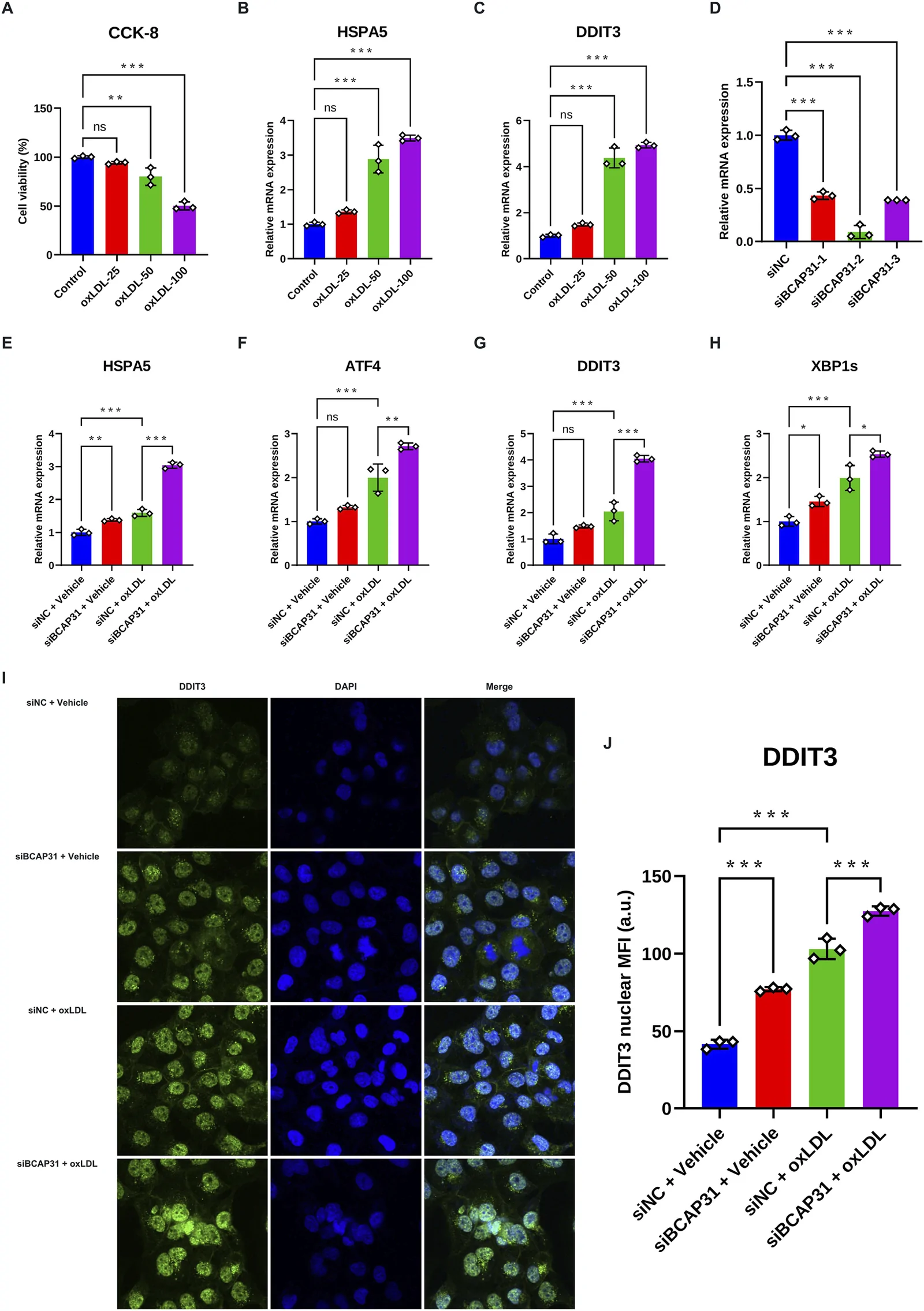
BCAP31 depletion amplifies oxLDL-induced endoplasmic reticulum stress. **(A)** CCK-8 viability after 24 h exposure to increasing oxLDL concentrations. **(B,C)** HSPA5 and DDIT3 mRNA across the concentration series. **(D)** BCAP31 mRNA after transfection with three candidate siRNAs; siBCAP31-2 was used subsequently. **(E–H)** HSPA5, ATF4, DDIT3, and XBP1s mRNA in siNC + vehicle, siBCAP31 + vehicle, siNC + oxLDL, and siBCAP31 + oxLDL groups. **(I)** Representative DDIT3, DAPI, and merged immunofluorescence images. **(J)** Nuclear DDIT3 mean fluorescence intensity. Data are shown as mean ± SD (n = 3 independent experiments). ns, not significant. Single, double, and triple asterisks denote P < 0.05, P < 0.01, and P < 0.001, respectively. Ordinary one-way ANOVA with Šídák adjustment was applied to three comparisons per endpoint. For A–C, adjusted P values for control versus 25, 50, and 100 μg/mL oxLDL, respectively, were **(A)** 0.479, 0.00368, 5.53e-06; **(B)** 0.18, 1.12e-05, 1.33e-06; **(C)** 0.0898, 2.47e-07, 7.5e-08. For D, siNC versus siBCAP31-1, −2, and −3 gave 5.38e-07, 1.3e-08, 3.03e-07, respectively. For E–H and J, the comparison order was siNC + vehicle versus siBCAP31 + vehicle; siNC + vehicle versus siNC + oxLDL; and siNC + oxLDL versus siBCAP31 + oxLDL. Adjusted P values in that order were **(E)** 0.00208, 9.13e-05, 1e-07; **(F)** 0.12, 0.000214, 0.00201; **(G)** 0.0769, 0.000934, 8.4e-06; **(H)** 0.0311, 0.000262, 0.0121; **(J)** 1.26e-05, 1.81e-07, 0.000196.

### 3.10 *BCAP31* depletion intensifies inflammatory output and apoptosis

*BCAP31* depletion increased the oxLDL-associated expression of *TNF*, *IL1B*, and *IL6* (Figures 10A–C). Culture-supernatant TNF-alpha, IL-1beta, and IL-6 showed concordant increases after normalization to cell number (Figures 10D–F), linking transcript changes to secreted inflammatory output. Annexin V/PI cytometry showed more apoptotic cells after oxLDL and a further increase in the *BCAP31*-depleted oxLDL group (Figures 10G,H). A smaller increase after *BCAP31* depletion under vehicle conditions paralleled the basal ER-stress changes. Thus, reducing *BCAP31* did not protect cells from an atherogenic challenge; it shifted ER stress, inflammation, and apoptosis in the same adverse direction.

**FIGURE 10:**
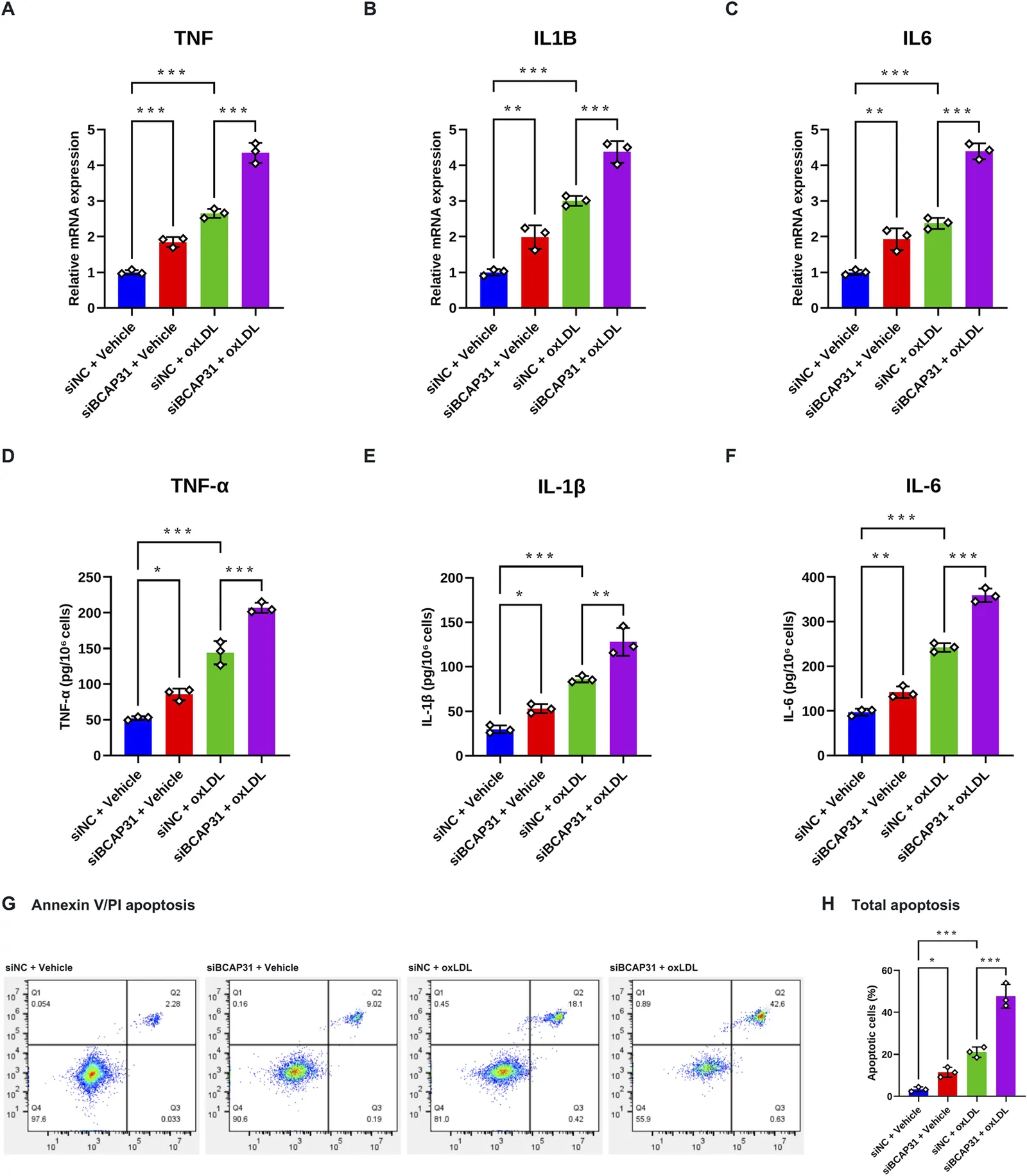
BCAP31 depletion increases inflammatory output and apoptosis. **(A–C)** TNF, IL1B, and IL6 mRNA in the four indicated groups. **(D–F)** TNF-alpha, IL-1beta, and IL-6 concentrations in culture supernatants normalized to cell number. **(G)** Representative Annexin V/PI flow-cytometry plots for the four groups. **(H)** Total apoptotic cells calculated as early plus late apoptotic populations. Data are shown as mean ± SD (n = 3 independent experiments). Single, double, and triple asterisks denote P < 0.05, P < 0.01, and P < 0.001, respectively. Ordinary one-way ANOVA with Šídák adjustment was applied to three comparisons per endpoint. The comparison order was siNC + vehicle versus siBCAP31 + vehicle; siNC + vehicle versus siNC + oxLDL; and siNC + oxLDL versus siBCAP31 + oxLDL. Adjusted P values in that order were **(A)** 0.000946, 7.73e-06, 6.35e-06; **(B)** 0.00296, 2.16e-05, 0.000342; **(C)** 0.00166, 0.000113, 6.38e-06; **(D)** 0.0101, 9.93e-06, 0.000161; **(E)** 0.034, 0.000141, 0.00109; **(F)** 0.00474, 1.05e-06, 5.49e-06; **(H)** 0.0496, 0.000543, 2.86e-05.

### 3.11 Chemical attenuation of ER stress partially reverses the *BCAP31*-depletion phenotype

To test whether the amplified inflammatory and apoptotic phenotype was linked to ER stress, BCAP31-depleted, oxLDL-exposed macrophages were treated with 4-PBA. 4-PBA reduced HSPA5, ATF4, DDIT3, and XBP1s relative to the BCAP31-depleted oxLDL group (Figures 11A–D). It also lowered supernatant TNF-alpha, IL-1beta, and IL-6 (Figures 11E–G) and reduced total Annexin V-positive apoptosis (Figures 11H,I). The concordant attenuation across ER-stress, cytokine, and apoptosis assays supports involvement of ER stress in the phenotype associated with BCAP31 depletion.

**FIGURE 11:**
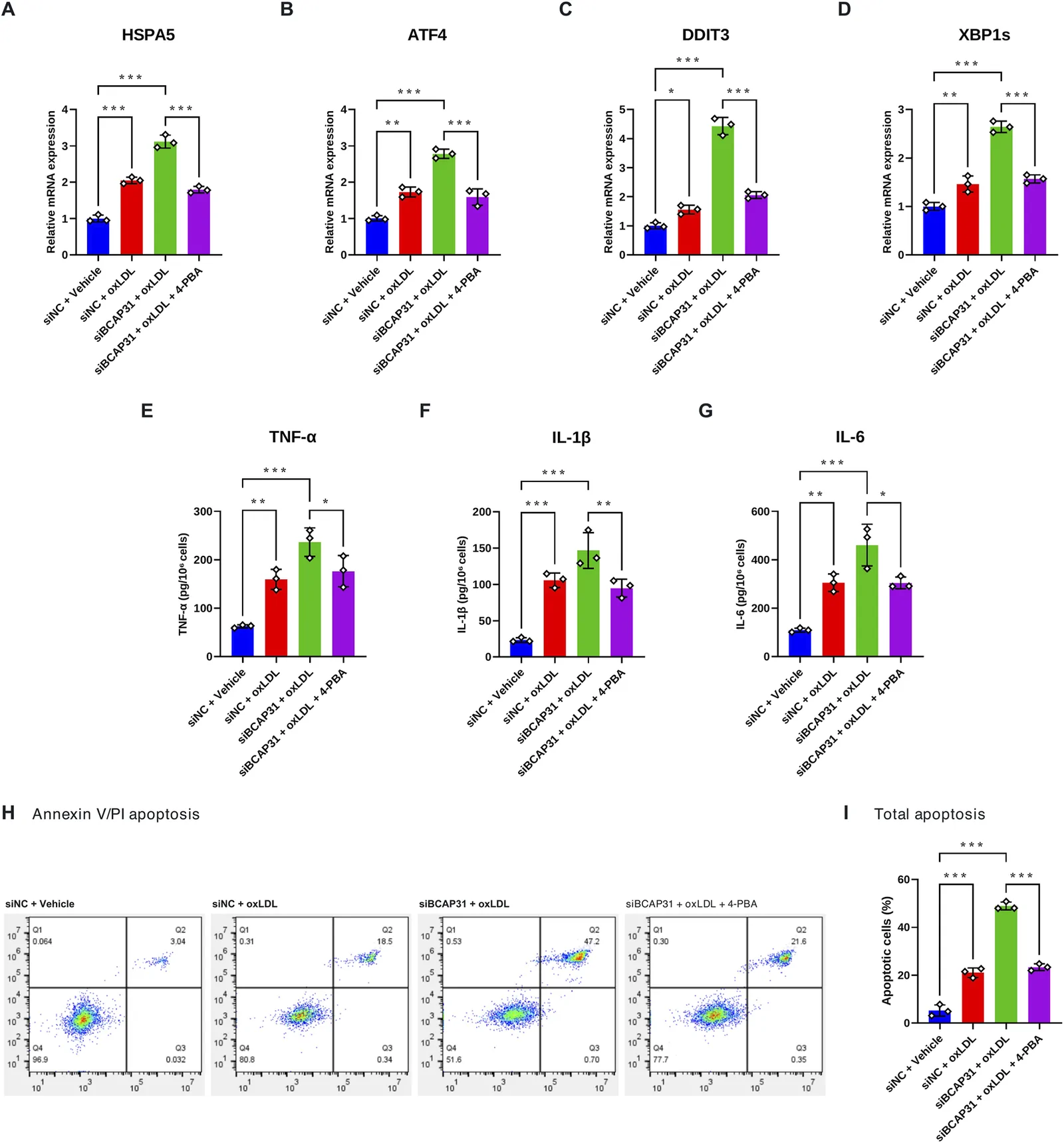
4-PBA attenuates the ER-stress-associated phenotype after BCAP31 depletion. **(A–D)** HSPA5, ATF4, DDIT3, and XBP1s mRNA in siNC + vehicle, siNC + oxLDL, siBCAP31 + oxLDL, and siBCAP31 + oxLDL + 4-PBA groups. **(E–G)** TNF-alpha, IL-1beta, and IL-6 concentrations in normalized culture supernatants. **(H)** Representative Annexin V/PI plots for the four groups. **(I)** Total apoptotic cells. Data are shown as mean ± SD (n = 3 independent experiments). Single, double, and triple asterisks denote P < 0.05, P < 0.01, and P < 0.001, respectively. Ordinary one-way ANOVA with Šídák adjustment was applied to three comparisons per endpoint. The comparison order was siNC + vehicle versus siNC + oxLDL; siNC + vehicle versus siBCAP31 + oxLDL; and siBCAP31 + oxLDL versus siBCAP31 + oxLDL + 4-PBA. Adjusted P values in that order were **(A)** 1.46e-05, 6.42e-08, 2.51e-06; **(B)** 0.00104, 1.44e-06, 3.04e-05; **(C)** 0.0178, 4.1e-08, 7.31e-07; **(D)** 0.00367, 3.8e-07, 9.9e-06; **(E)** 0.0036, 6.73e-05, 0.0485; **(F)** 0.000385, 2.03e-05, 0.00749; **(G)** 0.0034, 6.06e-05, 0.0124; **(I)** 2.02e-05, 7.38e-09, 5.01e-07.

## 4 Discussion

The combined human and cellular findings support a stress-adaptive interpretation of BCAP31 in carotid plaque biology. Its increased expression in plaque coexists with greater ER stress, inflammation, and apoptosis after depletion in oxLDL-exposed macrophages. Disease-associated elevation may therefore reflect an attempt to maintain cellular homeostasis rather than a simple pathogenic increase. This interpretation also accommodates the heterogeneity of the human data: whole-plaque transcription integrates several cellular processes, whereas macrophage and cell-state analyses identify the contexts in which a candidate may act.

The difference between tissue-level and cell-type-level signals is biologically plausible. Human plaques contain immune cells, VSMCs, endothelial cells, fibroblasts, extracellular matrix, necrotic material, and hemorrhage-related components whose proportions change with disease (Bentzon et al., 2014; Moore and Tabas, 2011). Bulk enrichment may reflect both within-lineage activation and expansion or loss of particular cellular compartments. Foundational single-cell studies established extensive macrophage and immune heterogeneity in atherosclerosis (Cochain et al., 2018; Winkels et al., 2018; Vallejo et al., 2021). Integrative work by Zhang et al. (2023) similarly approached plaque vulnerability through multiple molecular and clinical dimensions. Our results favor a model in which vulnerability emerges from several cell- and phenotype-specific stress responses, rather than from a uniform increase in one death program.

Plaque-enriched APOC1-SPP1-remodeling and SPP1-lipid-loaded macrophages provide a cellular context for the BCAP31 association. Such heterogeneity is consistent with macrophage and immune-state diversity described in single-cell atherosclerosis studies (Cochain et al., 2018; Winkels et al., 2018; Vallejo et al., 2021). In periodontitis, combined bulk and single-cell analysis likewise localized oxidative-stress-associated signatures to monocyte states (Song et al., 2023), underscoring the value of resolving stress programs within their cellular context. BCAP31 expression along the fitted ordering and predicted SPP1 interactions with vascular-wall cells suggest that intracellular stress adaptation accompanies tissue remodeling. The three-patient pseudobulk result remains directional evidence rather than an FDR-supported macrophage-specific association.

The direction of the perturbation experiments is central to interpretation. BCAP31 transcript abundance was higher in vulnerable human plaque contexts, yet BCAP31 depletion worsened ER stress, cytokine output, and apoptosis. This pattern is more consistent with a compensatory or stress-buffering response than with BCAP31 upregulation acting as a simple pathogenic driver. The unfolded protein response is adaptive at first and becomes death-promoting when proteostasis cannot be restored (Hetz, 2012; Ron and Walter, 2007; Ren et al., 2021; Tabas, 2010; Yang et al., 2020). BAP31 is positioned at the ER membrane and ER-mitochondria interface, where it participates in protein quality control and apoptotic signal transmission (Quistgaard, 2021; Iwasawa et al., 2011; Namba et al., 2013). Increased BCAP31 in stressed plaque macrophages may therefore mark greater demand for ER homeostasis. Its depletion was accompanied by higher DDIT3 expression, apoptosis, and inflammatory output, consistent with reduced stress-buffering capacity. A complementary macrophage-protective mechanism was demonstrated by Xu et al. (2025), who linked KIF13 B to MERTK-dependent efferocytosis in mice. That work highlights the distinction between preventing macrophage death and clearing cells that have already died; efferocytosis was not measured here.

This adaptive interpretation must be reconciled with the dual molecular forms of BAP31. Full-length, caspase-resistant BAP31 can inhibit Fas-associated mitochondrial events, whereas p20BAP31 promotes ER calcium release, mitochondrial fission, and cytochrome-c release (Nguyen et al., 2000; Breckenridge et al., 2003). Our transcriptomic datasets and qPCR assays measure BCAP31 mRNA and cannot determine protein abundance, cleavage, localization, or the full-length-to-p20 ratio. The present experiments therefore support a protective function of BCAP31 expression under the tested conditions but do not exclude pro-death signaling after BAP31 cleavage in advanced lesions. Immunoblotting with cleavage-state resolution would be required to close that mechanistic gap.

The attenuation by 4-PBA connects the BCAP31-depletion phenotype with ER proteostasis. ER stress contributes to advanced plaque macrophage apoptosis (Linton et al., 2016; Myoishi et al., 2007; Thorp et al., 2009; Tabas, 2010), and oxLDL-associated ER-stress responses can promote apoptosis and foam-cell formation (Zhao et al., 2016; Yao et al., 2014). Experimental ER-stress suppression by 4-PBA has also improved plaque-stability features in an atherosclerosis model (Zhu et al., 2024). Here, 4-PBA lowered ER-stress transcripts, cytokine release, and Annexin V positivity after BCAP31 depletion. In an osteoarthritis cell model, targeting the miR-140–3p/CILP axis reduced ferroptosis, oxidative stress, and inflammation (Ma et al., 2025); the different death pathway in that setting emphasizes that shared inflammatory readouts do not establish a shared mechanism. Because it is a broad chemical chaperone and the design lacked an oxLDL + 4-PBA group with intact BCAP31, these findings cannot separate general pharmacological protection from BCAP31-specific reversal. They support ER-stress involvement, not pathway-specific rescue or definitive epistasis.

The single-cell cohort contained three matched patients and the rupture cohort contained 11 patients, limiting power and generalizability. Whole-plaque associations may partly reflect cellular composition, while cross-sectional ordering and CellChat remain model-based descriptions. PMA-differentiated THP-1 cells do not reproduce the ontogeny or multicellular environment of primary plaque macrophages. Genetic re-expression and validation in primary human macrophages would help establish how broadly the observed stress-adaptive phenotype applies.

## 5 Conclusion

Patient-matched and instability-focused transcriptomics identified BCAP31 within a macrophage-associated stress context in human carotid atherosclerosis. BCAP31 depletion intensified oxLDL-induced ER stress, inflammatory cytokine production, and apoptosis, whereas 4-PBA attenuated these changes. These findings support a stress-adaptive role for BCAP31 under the tested conditions and motivate further investigation of its protein-level regulation in plaque macrophages.

## Data Availability

The original contributions presented in the study are included in the article/Supplementary Material, further inquiries can be directed to the corresponding authors.
